# Physical activity level and its sociodemographic correlates in a peri-urban Nepalese population: a cross-sectional study from the Jhaukhel-Duwakot health demographic surveillance site

**DOI:** 10.1186/1479-5868-11-39

**Published:** 2014-03-14

**Authors:** Abhinav Vaidya, Alexandra Krettek

**Affiliations:** 1 Department of Community Medicine, Kathmandu Medical College, Kathmandu, Nepal; 2 Department of Internal Medicine and Clinical Nutrition, Institute of Medicine, Sahlgrenska Academy at University of Gothenburg, Gothenburg, Sweden; 3 Nordic School of Public Health NHV, Gothenburg, Sweden

**Keywords:** Cardiovascular disease, Ethnicity, Occupation, Smoking, Hypertension, Diabetes

## Abstract

**Background:**

Physical inactivity is a leading risk factor for cardiovascular and other noncommunicable diseases in high-, low- and middle-income countries. Nepal, a low-income country in South Asia, is undergoing an epidemiological transition. Although the reported national prevalence of physical inactivity is relatively low, studies in urban and peri-urban localities have always shown higher prevalence. Therefore, this study aimed to measure physical activity in three domains—work, travel and leisure—in a peri-urban community and assess its variations across different sociodemographic correlates.

**Methods:**

Adult participants (n = 640) from six randomly selected wards of the Jhaukhel-Duwakot Health Demographic Surveillance Site (JD-HDSS) near Kathmandu responded to the Global Physical Activity Questionnaire. To determine total physical activity, we calculated the metabolic equivalent of task in minutes/week for each domain and combined the results. Respondents were categorized into high, moderate or low physical activity. We also calculated the odds ratio for low physical activity in various sociodemographic variables and self-reported cardiometabolic states.

**Results:**

The urbanizing JD-HDSS community showed a high prevalence of low physical activity (43.3%; 95% CI 39.4–47.1). Work-related activity contributed most to total physical activity. Furthermore, women and housewives and older, more educated and self-or government-employed respondents showed a greater prevalence of physical inactivity. Respondents with hypertension, diabetes or overweight/obesity reported less physical activity than individuals without those conditions. Only 5% of respondents identified physical inactivity as a cardiovascular risk factor.

**Conclusions:**

Our findings reveal a high burden of physical inactivity in a peri-urban community of Nepal. Improving the level of physical activity involves sensitizing people to its importance through appropriate multi-sector strategies that provide encouragement across all sociodemographic groups.

## Introduction

With an estimated prevalence of 31%, physical inactivity is the fourth leading cause of death worldwide [1], contributing to premature death (10%), coronary artery disease (6%) and type 2 diabetes mellitus (7%) [2]. In 1953, Jeremy Morris pioneered the epidemiology of physical inactivity by comparing the mortality rate for coronary heart disease (CHD) in bus drivers versus more active bus conductors [3]. Since the American Heart Association recognized physical inactivity as a risk factor for CHD in 1992 [4], longitudinal studies have further consolidated the protective role of physical activity against cardiovascular diseases (CVDs), including CHD and hypertension [5,6]. Consequently, public health organizations have initiated global efforts to curtail and counteract physical inactivity [7,8].

In general, physical inactivity occurs more commonly in high-income countries than low-income countries [9]. However, physical inactivity varies greatly between different regions of the World Health Organization (WHO), ranging from 17% in Southeast Asia to 43% in the Americas [9]. A recent review showed variation between countries in the Asia-Pacific region and even between studies in the same country [10]. Most notably, the prevalence of physical inactivity in Nepal ranges from a modest 8% to a staggering 82% [10].

The Nepal data [10] show how different measurement tools can yield varying results. Currently, standard tools include the Global Physical Activity Questionnaire (GPAQ) and the International Physical Activity Questionnaire (IPAQ). Mostly used in risk factor surveillance such as the WHO Stepwise Approach to Surveillance (STEPS) surveys [11], the GPAQ emerged as a compromise between the long and short versions of the IPAQ, which underwent several metamorphoses during the late 1990s [1,12-14]. The concept of domain in physical activity has evolved over time, shifting from an initial focus on leisure-time activity alone to its current emphasis on total physical activity (TPA), which includes leisure-time, occupational, housework and transport-related activity [9]. Further, objective measurement of physical activity with motion sensors and data comparison with questionnaire methods is increasingly a topic of interest and debate [15-18].

Apart from attempts to use standardized questionnaires to measure physical activity in different populations, recent longitudinal and cross-sectional studies have focused on determinants (i.e., variables with causal association) and correlates (i.e., variables with statistical association only), respectively [19]. Many determinants involve demographic, psychosocial, behavioral and social factors that reveal the innate complexity of physical inactivity [19]. Environmental (e.g., walkability) and policy (e.g., cycling policy) correlates influence an individual’s physical activity behavior [19,20].

Nepal, a small agrarian republic in South Asia with 26 million inhabitants, has conducted limited research on physical activity. However, earlier studies, including the nationwide WHO STEPs survey (2007–2008), did not investigate the sociodemographic correlates of physical inactivity [21-23]. In this regard, we previously highlighted the problem of increased CVD and risk factors in Nepal [24] and elaborated the impact of urbanization on changing lifestyle, including rising body mass index and declining levels of physical activity [25]. For example, although the current percentage of overweight in Nepal is not remarkably high (7%), it is definitely increasing [24]. Only 6% of urban males have CHD [26], but the prevalence of hypertension in Nepal’s peri-urban communities has tripled, from 6% to 18%, in 25 years [27]. Likewise, dyslipidemia and diabetes mellitus have been reported in 10% and 19% of urban Nepalese adults, respectively [28,29]. We previously suggested that health promotion might improve heart-healthy behaviors, including physical activity, and provide a starting point for CVD control in a resource-challenged country like Nepal [30].

However, when targeting various population subsets for positive behavioral changes such as physical activity, it is important to first explore how different sociodemographic factors influence activity patterns. Therefore, the current study in a peri-urban community of the Kathmandu valley aimed to conduct a detailed analysis of physical activity. We also sought to determine the relationship between physical activity and other behavioral and biological risk factors for cardiometabolic diseases and how it correlates with various sociodemographic factors.

## Methods

### Study site and population

We conducted the present study in the Jhaukhel-Duwakot Health Demographic Surveillance Site (JD-HDSS) [31]. Located 13 kilometers from Kathmandu in the Bhaktapur district of the Kathmandu valley, the two adjacent villages of JD-HDSS are rapidly transforming into peri-urban settlements. According to our 2010 baseline census, JD-HDSS includes 2,712 households and 13,669 inhabitants [31].

The terrain in JD-HDSS slopes from north to south. Although roadways connect the villages to the newly expanded six-lane Kathmandu-Bhaktapur highway, interior sections are connected only by narrow walking trails that increasingly are used for bicycles and motorbikes. Because cars and public vehicles are rare in JD-HDSS, young people, especially males, usually travel on motorbikes. Previously inhabited mostly by farmers, the agro-base of JD-HDSS is declining as more people shift to non-agrarian jobs. The three major ethnic groups are Brahmin, Chhetri and Newar. Common morbidities include respiratory diseases, resulting mainly from the high rate of tobacco smoking and brick factory smoke, and cardiometabolic disorders such as hypertension and diabetes mellitus [31].

### Data collection

After randomly selecting three wards (i.e., administrative units) from both Duwakot and Jhaukhel, we used our 2010 census data to prepare a list of all adults aged 25–59 years in all six wards. We selected one respondent from each household and applied the Kish technique [32] when a household contained more than one eligible candidate.

Twelve trained enumerators visited households in the selected wards between September and November 2011, conducting face-to-face interviews and recording physical measurements and blood pressures. The enumerators used a Microlife BR-9201 weighing machine (Microlife AG Swiss Corporation, Widnau, Switzerland) for weight measurement and non-stretchable tapes (Jonson Tapes Ltd, New Delhi, India) to determine height, waist and hip circumference. Blood pressure was measured digitally three times at 5-minute intervals, using Microlife BP 3AP1-3E (Microlife AG Swiss Corporation, Widnau, Switzerland). The final blood pressure measurement was an average of the three readings. In accordance with cutoffs established by the Joint National Committee–VII, we defined hypertension to include individuals with a known history of hypertension and also those diagnosed with hypertension during the survey (systolic blood pressure ≥ 140 mm Hg or diastolic blood pressure ≥ 90 mm Hg) [33]. Four public health graduates supervised the enumerators and a field coordinator and PhD student oversaw all data collection.

We used GPAQ version 2 to elicit information about three domains of physical activity: work (including housework), travel to and from places and leisure time [34]. Enumerators asked respondents how many days/week and the time/day they spent doing vigorous (e.g., lifting heavy loads) and moderate (e.g., carrying light loads) activities at work. They also asked respondents if they walked or cycled continuously for at least 10 minutes during their commute to work, market, etc. Questions about leisure-time physical activity included vigorous (e.g., intense sports) and moderate activity (e.g., swimming). In accordance with the GPAQ Analysis Guide, we converted the responses to metabolic equivalent to task (MET)–minutes/week [34]. Further, we categorized respondents’ physical activity as high, moderate or low depending on their total MET–minutes/week or other combination criteria [34]. This paper presents the prevalence of low physical activity (LPA) as an indicator of insufficient physical activity. In addition, we sorted respondents according to the WHO-recommended minimum of 150 minutes of moderate or 75 minutes of vigorous aerobic physical activity throughout the week [35].

We categorized sociodemographic variables in accordance with the WHO STEPS manual [11] and the classifications of the Central Bureau of Statistics, Nepal [36]. Our definition of primary and secondary education included respondents who studied up to grades 4 (usually aged 10 years) and 10 (usually aged 16 years), respectively. We based our categorization of smoking and alcohol consumption on the WHO STEPS manual [11], defining respondents who replied “yes” to “Do you smoke?” as current smokers and smokers who replied “yes” to “Did you ever smoke in the past?” as past smokers. Current drinkers had consumed alcohol within the previous month.

### Data analysis

Data entry operators recorded data in Epidata version 2.1 and we analyzed the data using SPSS version 17.0 (IBM, Armonk, New York, USA) and STATA version 10.0 (StataCorp, Texas, USA). Our results present categorical values as percentages and the continuous variable as median. We conducted correlation analysis and calculated Spearman’s correlation coefficient for continuous variables. Our results report the unadjusted and adjusted odds ratios (OR) for LPA compared to moderate-to-vigorous physical activity, a single category created by combining the moderately and vigorously active groups. ORs were calculated for the various sociodemographic and cardiovascular risk factor correlates.

### Ethical considerations

Our study was approved by the Nepal Health Research Council and the Institutional Review Board of Kathmandu Medical College. After briefing respondents about the purpose of the study, enumerators requested informed consent. Respondents were free to end the survey or skip any question or section. We maintained interview confidentiality by conducting surveys either indoors or in isolation. Hard copies of the data were stored securely in the JD-HDSS office in Jhaukhel. Newly diagnosed hypertensives and respondents who needed urgent health care were referred to Kathmandu Medical College or Nepal Medical College Community Hospital, where they were entitled to subsidized service charges.

## Results

Among 840 households visited in 6 randomly selected wards, 789 individuals consented to participate in the study (93.9%). Among those, we obtained complete information on demographic variables from 777 respondents. We excluded respondents for which information on height, weight or blood pressure measurements was incomplete. Thus, final analyses were based on 640 individuals.

### Demographic background and risk factors status

Table 1 shows the sociodemographic composition of the study population. Females were unintentionally oversampled (72.7%) and the three major ethnic groups (i.e., Brahmin, Chhetri and Newar) were represented proportionally. One fifth of respondents smoked tobacco (males, 33%; females, 15%) or currently drank alcohol (males, 35%; females, 13%). One fifth of the respondents had high blood pressure (males, 22%; females, 21%). Female respondents had more generalized (11% vs. 5% in males) and abdominal (56% vs. 21% in males) obesity.

**Table 1:** Percentage (95% confidence interval) of population meeting the WHO recommendation for physical activity, and those categorized as having low, moderate and vigorous activity according to the GPAQ classification

	**N**	**Population meeting WHO recommendation for physical activity (%)**		**GPAQ classification**					
				**Low physical activity (%)**		**Moderate physical activity (%)**		**Vigorous physical activity (%)**	
		**Male (n = 175)**	**Female (n = 465)**	**Male (n = 175)**	**Female (n = 465)**	**Male (n = 175)**	**Female (n = 465)**	**Male (n = 175)**	**Female (n = 465)**
**All**	640	82.1 (77.1–87.0)	78.1 (74.6– 81.5)	38.3 (31.0– 45.5)	45.1 (40.6– 49.7)	56.5 (49.2– 63.9)	48.6 (44.0– 53.2)	5.1 (1.8– 8.4)	6.2 (4.0– 8.4)
**Age (years)**									
45–59	189	82.9 (74.7–91.1)	70.2 (63.1–77.4)	37.3 (24.8–49.7)	49.2 (40.6–57.9)	61.0 (48.4–73.6)	46.1 (37.5–54.8)	1.7 (1.6–5.0)	4.6 (0.9–8.2)
35–44	240	82.1 (73.4–90.6)	84.6 (79.6–89.6)	38.1 (25.9–50.2)	44.0 (36.7–51.4)	53.9 (41.5–66.4)	48.6 (41.2–55.9)	7.9 (11.9–14.6)	7.3 (3.5–11.2)
25–34	211	81.1 (71.8– 90.5)	77.7 (71.7–83.6)	39.6 (26.3–52.9)	43.0 (35.3–50.8)	54.7 (41.2–68.3)	50.6 (42.8–58.5)	5.6 (0.6–11.9)	6.3 (2.5–10.1)
**Ethnicity**									
Brahmin	232	85.9 (78.4–93.3)	67.5 (61.1–73.8)	29.2 (18.0–40.3)	49.1 (41.4–56.7)	63.1 (51.2–74.9)	43.7 (36.1–51.3)	7.6 (1.1–14.2)	7.2 (3.2–11.1)
Chhetri	170	74.5 (62.9–86.2)	82.7 (76.4–89.0)	46.6 (31.9–61.4)	49.6 (40.7–58.4)	53.3 (38.5–68.1)	44.8 (36.0–53.5)	0.0 (0.0–0.0)	5.6 (1.5–9.6)
Newar	167	86.5 (78.3–94.8)	88.2 (82.5–93.8)	33.3 (20.2–46.4)	36.2 (27.4–45.0)	62.7 (49.3–76.2)	59.5 (50.5–68.5)	3.9 (1.4–9.3)	4.3 (0.6–8.02)
Ethnic minorities*	71	72.7 (53.6–91.8)	82.2 (73.3–91.0)	71.4 (46.8–90.0)	42.1 (29.1–55.0)	14.3 (4.8–33.3)	49.1 (36.0–62.2)	14.3 (4.8–33.3)	8.8 (1.3–1.61)
**Education†**									
High school or more	94	68.5 (55.9–81.0)	67.1 (56.0–78.2)	51.3 (34.9–67.7)	70.2 (58.2–82.8)	48.6 (32.2–65.0)	26.3 (14.7–37.8)	0.0 (0.0–0.0)	3.5 (1.3–8.3)
Secondary	165	86.4 (78.6–94.3)	77.4 (70.0–84.8)	41.6 (29.0–54.3)	46.7 (37.1–56.3)	53.3 (40.6–66.1)	47.6 (38.0–57.2)	5.0 (0.5–10.5)	5.7 (1.2–10.1)
Primary	208	87.8 (80.3–95.3)	80.8 (74.9–86.7)	23.3 (12.5–34.1)	39.8 (31.9–47.7)	68.3 (56.4–80.2)	52.0 (43.9–60.1)	8.3 (1.3–15.4)	8.1 (3.6–12.5)
Informal	173	81.5 (66.5–96.4)	80.2 (74.4–86.0)	50.0 (26.2–73.8)	40.0 (32.2–47.7)	44.4 (20.8–68.1)	54.2 (46.3–62.1)	5.5 (5.3–16.4)	58.0 (2.1–9.5)
**Occupation‡**									
Government job	41	70.9 (54.6–87.2)	75.0 (53.0–96.9)	51.8 (32.6–71.1)	50.0 (22.7–77.2)	48.1 (28.9–67.3)	42.8 (15.9–69.8)	0.0 (0.0–0.0)	7.1 (6.8–21.1)
Nongovernment job	61	92.3 (83.8–100.0)	76.7 (61.2–92.1)	26.5 (11.4–41.5)	59.2 (40.3–78.2)	67.6 (51.6–83.6)	40.7 (21.8–59.6)	58.8 (2.1–13.9)	0.0 (0.0–0.0)
Self–employed	80	70.8 (60.2–81.4)	63.9 (47.9–79.8)	43.4 (29.9–56.9)	48.1 (28.9–67.3)	52.8 (39.2–66.4)	48.1 (28.9–67.3)	3.7 (1.4–8.9)	3.7 (3.5–10.9)
Housewife§	341	-	79.4 (75.2–83.5)	-	46.0 (40.5–51.4)	-	48.1 (42.7–53.6)	-	5.8 (3.2–8.3)
Agriculture	117	95.8 (90.1–100.0)	83.3 (74.9–91.6)	30.4 (16.9–43.9)	33.8 (22.7–44.9)	65.2 (51.2–79.1)	54.9 (43.2–66.6)	4.3 (1.6–10.3)	11.2 (3.8–18.6)
**Smoking**									
Current smoker	132	80.4 (68.8–92.0)	78.2 (70.4–85.9)	33.3 (21.2–45.4)	47.2 (35.5–58.8)	61.6 (49.2–74.0)	47.2 (35.5–58.8)	5.0 (0.5–10.5)	5.5 (0.2–10.9)
Past smoker	34	73.3 (50.1–96.5)	74.3 (59.6–88.9)	35.7 (9.6–61.8)	15.0 (1.0–31.0)	64.2 (38.2–90.4)	80.0 (61.9–98.0)	0.0 (0.0–0.0)	5.0 (0.5–14.8)
Nonsmoker	474	83.3 (77.6–88.9)	78.4 (74.4–82.4)	41.6 (31.9–51.2)	46.3 (41.3–51.4)	52.4 (42.6–62.3)	47.2 (42.1–52.2)	5.9 (1.3–10.6)	6.4 (3.9–8.9)
**Current drinker**									
Yes	118	84.8 (76.8–92.8)	89.8 (82.7–97.0)	26.7 (15.4–37.9)	34.4 (22.1–46.8)	0.70 (58.2–81.7)	60.3 (47.6–73.0)	3.3 (1.2–7.9)	5.2 (0.6–10.9)
No	522	80.6 (74.3–87.0)	76.4 (72.6–80.2)	44.3 (35.2–53.5)	46.7 (41.8–51.5)	49.6 (40.3–58.7)	46.9 (42.0–51.7)	6.0 (1.6–10.5)	6.4 (4.0–8.7)
**Diagnosed hypertension**									
Yes	94	79.3 (64.3–94.3)	75.3 (65.8–84.7)	52.4 (30.4–74.3)	52.0 (40.5–63.6)	42.8 (21.1–64.5)	42.5 (31.0–53.9)	4.7 (0.4–14.1)	5.4 (0.2–10.7)
No	546	82.5 (77.2–87.7)	78.6 (74.8–82.3)	36.4 (28.7–44.0)	43.8 (38.9–48.8)	58.4 (50.6–66.3)	49.7 (44.8–54.7)	5.2 (1.6–8.7)	6.3 (3.9–8.8)
**Diagnosed diabetes**									
Yes	26	81.8 (57.6–106.0)	73.7 (53.1–94.3)	60.0 (27.9–92.0)	50.0 (24.6–75.3)	40.0 (7.9–72.0)	50.0 (24.6–75.3)	0.0 (0.0–0.0)	0.0 (0.0–0.0)
No	614	70.9 (54.5–87.4)	77.5 (64.2–90.7)	36.9 (29.5–44.3)	44.9 (40.4–49.6)	57.6 (49.9–65.1)	48.5 (43.9–53.2)	5.4 (1.9–8.9)	6.4 (4.1–8.7)
**Body mass index**									
>25	248	69.8 (59.2–80.4)	78.7 (73.5–83.9)	53.8 (40.1–67.5)	50.5 (43.5–57.5)	44.2 (30.6–57.9)	44.4 (37.4–51.4)	1.9 (0.2–5.7)	5.1 (2.0–8.2)
<25	392	87.8 (82.6–92.9)	77.6 (72.9–82.2)	30.2 (21.9–38.5)	41.3 (35.4–47.3)	63.0 (54.3–71.7)	51.3 (45.2–57.4)	6.7 (21,2–11.2)	7.3 (4.1–10.4)
**Waist circumference**									
Increased	298	79.6 (68.2–91.0)	75.4 (70.6–80.2)	42.5 (26.9–58.0)	53.1 (46.9–59.2)	55.0 (39.3–70.6)	42.2 (36.2–48.3)	2.5 (.2–7.4)	4.6 (2.1–7.2)
Normal	342	83.1 (77.6–88.7)	82.2 (77.4–87.2)	37.3 (29.0–45.5)	35.2 (28.7–41.8)	56.7 (48.3–65.2)	56.5 (49.7–63.3)	5.9 (1.9–10.0)	8.2 (4.4–11.9)
**Waist–hip ratio**									
Increased	348	77.4 (68.8–85.9)	77.9 (73.1–82.6)	33.7 (24.1–43.2)	46.2 (0.40–0.52)	61.0 (51.2–70.9)	47.4 (41.2–53.6)	5.2 (0.7–9.7)	6.3 (3.3–9.3)
Normal	292	85.2 (78.9–91.3)	78.7 (73.4–83.9)	46.5 (35.0–58.1)	44.4 (37.5–51.4)	49.3 (37.7–60.8)	48.9 (41.9–55.9)	6.3 (3.3–9.3)	6.5 (3.1–10.0)

Note. GPAQ = Global Physical Activity Questionnaire; WHO = World Health Organization.

*Includes Rai, Magar, Tamang, Dalit, Gurung, Mandal, Chaudary, Pariayar, Purkutti.

†Primary education includes grade 1–4, secondary education includes grades 6–10, and high school begins with grade 11.

‡The definitions of occupation were adopted from the Nepal WHO–STEPS Non–Communicable Disease Survey, 2007 [21].

§A woman who is involved in her own household activities (e.g., cooking, washing, cleaning, etc.) but does not earn money.

Risk factor categories were based on the WHO–NCD Risk Factor STEPS Survey manual [11]. Current smokers included those who responded “yes” to “Do you smoke?” Past smokers included those who replied “yes” to “Did you ever smoke in the past?”. Current drinkers included respondents who had consumed alcohol within the previous month. Blood pressure data exclude respondents who did not submit to all three readings. Likewise, data for body mass index, waist circumference and waist–hip ratio exclude respondents whose weight, height and waist and/or hip measurements were not taken. Increased waist circumference includes waist measurements of ≥80 cm (women) and ≥90 cm (men); increased waist–hip ratio was ≥ 0.85 (women) and ≥0.90 (men).

### Domains of physical activity

We investigated physical activity level in three domains (i.e., work, travel and leisure) and combined the results to assess TPA (Table 1). Work-related activities contributed most to TPA, particularly in females, housewives, Newars, ethnic minorities and those with informal education (>90% of TPA). In general, travel and leisure-time physical activity (LTPA) contributed less to TPA. Travel-related activities were particularly less common among government employees, housewives and those involved in agricultural work. LTPA in males, Brahmins and employed individuals (governmental, nongovernmental or self-employed) accounted for around one quarter of TPA.

### Burden of physical inactivity

Based on the WHO guideline [35], 82.1% of males and 78.1% of females achieved the recommended weekly levels of minimal physical activity (Table 2). The proportion was highest among males working in agriculture (95.8%) and lowest among self-employed females (Table 2). However, the GPAQ classification showed that a larger proportion of our study population (i.e., 38.3% of males and 45.1% of females) had LPA. LPA was highest among ethnic minority males (71.4%) and lowest among males who had studied up to grade 4 (23.3%) (Table 2). Correspondingly, 50.8% of respondents demonstrated moderate or high physical activity (50.8% and 5.9%, respectively).

**Table 2:** Physical activity in different domains of activity and odds ratio for low physical activity compared to moderate to vigorous activity, according to the various demographic factors and risk factor status of the study population

	**N**	**Median physical activity (MET-minutes/week)**				**Unadjusted odds ratio for LPA vs. MVPA (95% CI)**	Adjusted odds ratio* for LPA vs. MVPA (95% CI)
		**Work time**	**Travel time**	**Leisure time**	**Total**		
**All**	640	384	36	36	456	-	-
**Sex**							
Female	465	432	29	14	475	1.33 (0.93–1.88)	1.75 (1.19–2.54)
Male	175	288	76	144	508	1	1
**Age (years)**							
45–59	189	288	25	29	342	1.14 (0.77–1.70)	1.67 (1.08–2.58)
35–44	240	456	46	35	536	1.01 (0.69–1.47)	1.18 (0.80–1.75)
25–34	211	432	43	48	523	1	1
**Ethnicity**							
Brahmin	232	288	46	83	417	0.84 (0.49–1.43)	0.53 (0.29–0.94)
Chhetri	170	334	96	43	473	1.04 (0.59–1.80)	0.86 (0.48–1.52)
Newar	167	576	10	24	610	0.59 (0.34–1.04)	0.57 (0.31–0.97)
Ethnic minorities	71	432	22	0	454	1	1
**Education**							
High school or further	94	180	24	14	218	2.42 (1.44–4.07)	2.99 (1.65–5.46)
Secondary	165	336	50	96	482	1.17 (0.76–1.80)	1.42 (0.86–2.32)
Primary	208	492	49	72	613	0.78 (0.51–1.17)	0.86 (0.55–1.33)
Informal	173	576	24	0	600	1	1
**Occupation**							
Government job	41	192	24	55	271	2.18 (1.06–4.50)	1.97 (0.88–4.42)
Nongovernment job	61	288	144	144	576	1.44 (0.76–2.74)	1.16 (0.58–2.32)
Self-employed	80	288	46	96	430	1.70 (0.94–3.06)	1.76 (0.95–3.25)
Housewife	341	432	24	0	456	1.77 (1.14–2.76)	1.59 (0.99–2.56)
Agriculture	117	576	50	72	698	1	1
**Smoking**							
Current smoker	132	348	49	50	447	0.83 (0.56–1.23)	1.03 (0.67–1.59)
Past smoker	34	492	47	295	834	0.37 (0.16–0.83)	0.43 (0.18–0.99)
Nonsmoker	474	384	29	16	429	1	1
**Current drinker**							
Yes	118	528	28	84	640	0.51 (0.33-0.78)	0.56 (0.35-0.91)
No	522	372	36	25	433	1	1
**Diagnosed hypertension**							
Yes	94	372	14	8	394	1.52 (0.98–2.35)	1.41 (0.88–2.23)
No	546	384	48	48	480	1	1
**Diagnosed diabetes**							
Yes	26	334	8	0	342	1.56 (0.71–3.42)	1.64 (0.73–3.67)
No	614	384	36	43	463	1	1
**BMI**							
>25	248	288	29	0	317	1.72 (1.24–2.38)	1.58 (1.13–2.20)
<25	392	432	48	96	576	1	1
**Waist**							
Increased	298	336	29	0	365	1.89 (1.38–2.60)	1.78 (1.27–2.49)
Normal	342	432	48	96	576	1	1
**Waist-hip ratio**							
Increased	348	336	46	50	432	0.91 (0.66–1.23)	0.91 (0.65–1.26)
Normal	292	504	29	0	533	1	1

Note. LPA = low physical activity; MET = metabolic equivalent to task; MVPA = moderate to vigorous activity.

*Odds ratios for sex, age, ethnicity, education and occupation were adjusted for sociodemographic variables other than the dependent variable itself. Odds ratios for risk factor variables were adjusted for sociodemographic variables (i.e. age, sex, ethnicity, education and occupation).

### Sociodemographic correlates of physical activity

We also analyzed whether physical activity associates with sociodemographic factors such as gender, age, ethnicity, education and occupation (Table 1). Although males had higher total MET–minutes/week than females, women reported a higher rate of work-related physical activity (Table 1). Nonetheless, female respondents were 1.75 times more likely than males to have an inadequate level of physical activity as defined by the GPAQ (Table 1). LPA was greatest among the oldest age group, especially females, in all domains (adjusted OR = 1.67 [95% CI 1.08–2.58]) (Table 1). Our results show a negative correlation between age and physical activity during work, travel, leisure and TPA (Spearman’s correlation coefficient: -0.096, -0.053, -0.019 and -0.078, respectively).

Ethnic variations were evident. Newars reported more work-related activity and higher TPA, whereas Brahmins reported more physical activity during leisure time (Table 1). Ethnic minorities showed less physical inactivity. Compared with informal education, physical inactivity was three times higher in individuals educated up to high school or more (Table 1). Respondents who worked in agro-based jobs had the highest TPA (Table 1). In terms of TPA, inadequate physical activity was more likely in government employees, self-employed individuals and housewives (Table 1).

### Association of physical activity with cardiovascular risk factors

Compared to individuals with no risk factors for CVD, respondents diagnosed with hypertension, diabetes mellitus or overweight/obesity showed a higher prevalence and increased odds for LPA (Table 1 and Table 2).

## Discussion

### Insufficient physical activity in Nepal typical of a low-income nation

The prevalence of LPA in our peri-urban study population is consistent with the findings of smaller studies in urban Nepal [22,23] but less compared to studies in the neighboring capital city of Kathmandu (82%) [21]. However, this prevalence far exceeds the national average (5.5% [95% CI 3.4–7.7]) reported by the WHO STEPs survey using GPAQ [21] or the 8% estimated by the World Health Survey using IPAQ [37].

A comparison of Nepal to other countries clearly demonstrates worldwide variations in the prevalence of physical inactivity [2,9]. Generally, the prevalence of physical activity associates positively with the national economy, ranging from around 5% in Bangladesh (another low-income nation) to around 15% in middle-income countries such as India and Viet Nam and greater prevalence in high-income countries such as Australia (38%), the United States of America (40%) and the United Kingdom (63%) [9,38]. In addition, studies from other Asian HDSSs, including Vietnam (e.g., 13% in the rural Chililab HDSS and 58% in the urban Filabavi HDSS) show rural–urban disparities in physical inactivity levels [39]. Similarly, the current burden of physical inactivity in Nepal clusters around urban and urbanizing populations [40].

### Contrasting physical activity in different domains

Despite rapidly declining levels of physical activity worldwide [41], emerging evidence suggests that different domains, particularly leisure time, play an important role in CHD reduction [42,43]. Active transportation methods (e.g., cycling and walking) associate with decreased levels of all-cause mortality [9]. Further, some studies have explored the interrelationship between different domains of physical activity. For example, we found a positive correlation between occupational physical activity and LTPA, results that concur with a study in the United States [44]. However, our study did not investigate other occupation-related factors that inversely associate with leisure activity (e.g., job strain, working hours and overtime) [19].

Our finding of less physical activity during leisure time concurs with results from other urban [45] and HDSS studies in Asia [39]. In the context of traditionally urban Nepal, leisure-time activities (e.g., sports and exercise, including jogging) associate more frequently with youth or modern culture. Most Nepalese spend their leisure time watching television, socializing, gossiping or playing cards. Moreover, physical activity levels in Nepal vary seasonally and physiologically [46,47].

Like many low-income countries, most physical activity in Nepal associates with work or occupation-related activities [19]. In contrast, high-income countries exhibit lower work-related physical activity, thus encouraging physical activities during transportation (e.g., cycling in Denmark) and leisure (e.g., Sweden, Canada, England and Spain) [9].

### Sociodemographic variations in physical activity levels

In the present study, women did more household chores than men, reflecting an almost universal pattern in the traditionally patriarchal society in Nepal and other Asian countries [39,45]. Nonetheless, our female respondents were more likely to have low overall physical activity. Male sex is a positive determinant of greater physical activity in children aged 4–9 years but not thereafter [19]. Further, risk reduction for CHD through regular physical activity is more pronounced in women than men (40% and 30%, respectively) [20]. In terms of age, the inverse relationship between age and physical activity is an almost global phenomenon, with some notable exceptions (i.e., New Zealand, Australia, China and some East Asian countries) [20,48,49]. Importantly, physical activity reduces cardiovascular risk even in old age [20].

In the present study, ethnic minorities showed less physical inactivity, a result that concurs with other findings [48,50,51] including those by studies that conducted objective measurements in children of different ethnic background [52]. Earlier studies attribute this disparity to fewer facilities for outdoor physical activity and a greater number of fast-food outlets in ethnic neighborhoods [53]. This discrepancy persists even after adjusting for possible sociodemographic variables, health-related factors and health-belief variations [54]. Further, physical activity preference differs according to ethnicity [55]. Indeed, protection against CVD through physical activity is apparent across not only gender and age but also ethnicity [20].

Studies in developed countries report that education level correlates positively with physical activity [19,48], but our results revealed a step-wise inverse relationship. Other Asian HDSSs report similar findings [39], suggesting the probability of a contrasting trend of physical activity across the educational strata in low- and high-income countries. Our finding of highest TPA but lowest LTPA among agricultural workers concurs with findings from India [56], China [57], Finland [58] and elsewhere [19].

### Lower level of physical activity in people with cardiovascular risk factors

Our respondents showed a similar prevalence of smoking, and current alcohol consumption was lower than the national average [21]. The prevalence of hypertension was similar to the national percentage [21]. Obesity was double the national average [21] and similar to that in nearby urban Kathmandu [21], hinting that the effect of urbanization is spilling from the urban area into nearby rural communities, a phenomenon already demonstrated in India [59]. In our study, the probability of inadequate physical activity was higher among individuals with diagnosed hypertension, diabetes mellitus or overweight/obesity. Due to the cross-sectional nature of our study, we cannot comment on physical inactivity as a cause or consequence of these cardiometabolic conditions. Nonetheless, earlier studies report decreased levels of physical activity following diagnosis with a chronic condition such as diabetes [60] or hypertension [61], mainly due to co-morbidities (e.g., arthritis) or social demands [62]. This phenomenon has been observed worldwide, particularly in low-income countries [63]. In addition, the relationship between overweight/obesity and physical inactivity can create a vicious cycle where in various psychosocial factors (e.g., fear of being teased or bullied, fear of negative judgments and lack of social or peer support) limit physical activity in overweight/obese people in [64].

### Physical activity as an outcome of urbanization and changing lifestyle in Nepal

In our study population, high prevalence of physical inactivity reflects a side effect of development and urbanization in a low-income country like Nepal. For example, farming, which is a physically demanding occupation, decreased from 94% to 65% during the last 30 years [65]. This reduction corresponds with a similar decrease (from 60% to 33%) in agriculture’s share of GDP [65] and an escalating utilization of local land for new construction [66]. Further, although most farm equipment in Nepal is still powered by animals (41%) or humans (36%), mechanized equipment is gradually increasing (23%) [67]. Increased availability of motorized vehicles [68] and improved water supplies [69] have drastically decreased tradesmen’s demand for business-related mobility and women’s need to walk long distances for basic requirements (e.g., water). Modern technical gadgets that promote sedentary behavior frequently replace leisure activities (e.g., games and cultural rituals) [70], especially in the children and young adults.

Our results show that occupational activities comprise a major portion of total physical activity in JD-HDSS, raising concern for public health. Economic growth increases urbanization and reduces physical activity [1,71]. Earlier trends for physical activity transition [72] in western countries [73,74] and, more recently, in rapidly changing economies (e.g., China) [75] suggest that sedentary jobs will increase in Nepal. Lacking attempts to counteract this inevitable development by increasing physical activity in other domains (i.e., transport and leisure), overall physical activity likely will decline. Therefore, Nepal should not delay initiating interventions that improve physical activity through community-based strategies that incorporate informational, behavioral, social, policy and environmental approaches [76-78].

### Lack of physical activity-friendly environment in urban Nepal

Although built environments influence physical activity [79,80], our study did not explore this issue. Nepal’s roads are generally considered the most dangerous in the world for pedestrians [81-83], largely due to nonexistent pavement or cycling lanes, muddy and dilapidated roads, escalating traffic and congestion, negligent drivers who violate traffic rules, air pollution from dust and vehicular emissions and encroachment by street vendors. Many of these factors, including walkability [84], have a significant association with physical activity [85]. Further, Nepal’s urbanization is completely unplanned and settlements are haphazard. Parks and playgrounds, which associate positively with leisure-time physical activity in other settings [86,87] are uncommon in urban areas of Nepal. When governments do not prioritize aesthetic appeal and green space, people are less likely to engage in physical activity, especially during commuting and leisure time [19]. Contextually, the built environment has a complex relationship with psychosocial and sociocultural aspects of physical activity [79]. To identify targets for possible interventions to increase physical activity, future research should address these areas.

### Strengths and limitations of the study

Our study is the first detailed report on physical activity in an urbanizing population of Nepal. Earlier studies on the epidemiology of physical activity were conducted in high-income countries [9], but our timely study bridges this knowledge gap in a low-income setting. Similar to other HDSS studies [88], JD-HDSS provides a good platform for understanding the patterns of physical activity patterns in a peri-urban population. In addition, the longitudinal nature of an HDSS offers the advantage of systematic follow-up to examine trends and the effectiveness of interventions [89]. Moreover, our analysis demonstrates and reinforces the importance of ethnicity in cardiometabolic risk assessment [90-92]. However, our study did not explore in detail the social and environmental correlates of physical activity.

We adapted international data collection tools (including self-reported physical activity) to the local Nepali context [12,34]. Although self-reported questionnaires provide inadequate validity compared to objectively-measured physical activity tools such as accelerometers [15,93-95], such tools were too sophisticated, logistically impractical and expensive for successful use in our study setting. Additionally, objective tools carry their own limitations [9].

Despite the application of Kish technique during the selection of respondents at the household level, we previously reported unintentional oversampling of women [96]. We addressed this deficit by stratifying our results according to gender. Further, we excluded 137 of 777 respondents during analysis due to incomplete information regarding blood pressure or anthropometric measurements. Although including only respondents with complete information (i.e., three readings for each measurement) improved the validity of the study, the process also resulted in an 18% reduction in the study sample.

## Conclusions

This study investigated different domains of physical activity among adults in a peri-urban community of Nepal and explored their associations with various socioeconomic variables. Our respondents (especially government employees, self-employed individuals and housewives) showed a high prevalence of LPA. We also investigated the changing trend of physical activity in a low-income country and the effect of urbanization (e.g., decreased energy expenditure during work and travel and technology-driven leisure-time activities). Improving the level of physical activity in Nepal will require a multi-sector approach.

## Abbreviations

CHD: Coronary heart disease; CVD: Cardiovascular disease; GPAQ: Global Physical Activity Questionnaire; IPAQ: International Physical Activity Questionnaire; JD-HDSS: Jhaukhel-Duwakot Health Demographic Surveillance Site; LPA: Low physical activity; LTPA: Leisure-time physical activity; MET: Metabolic equivalent to task; NCD: Noncommunicable disease; TPA: Total physical activity; WHO: World Health Organization; WHO STEPS: WHO STEPwise approach to Surveillance.

## Competing interests

The authors declare that they have no competing interests.

## Authors’ contributions

AV designed the study, performed statistical analysis, and drafted the manuscript. AK helped design the study and provided critical revision of the manuscript. Both authors have read and approved the final manuscript.
